# Wound and Skin Healing in Space: The 3D Bioprinting Perspective

**DOI:** 10.3389/fbioe.2021.720217

**Published:** 2021-10-25

**Authors:** Nieves Cubo-Mateo, Michael Gelinsky

**Affiliations:** ^1^ Centre for Translational Bone, Joint and Soft Tissue Research, University Hospital Carl Gustav Carus and Faculty of Medicine, Technische Universität Dresden, Dresden, Germany; ^2^ Escuela Superior de Ciencia y Tecnología, Universidad Internacional de Valencia, Valencia, Spain

**Keywords:** wound healing, skin, bioprinting, space, microgravity, bioinks, biofabrication

## Abstract

Skin wound healing is known to be impaired in space. As skin is the tissue mostly at risk to become injured during manned space missions, there is the need for a better understanding of the biological mechanisms behind the reduced wound healing capacity in space. In addition, for far-distant and long-term manned space missions like the exploration of Mars or other extraterrestrial human settlements, e.g., on the Moon, new effective treatment options for severe skin injuries have to be developed. However, these need to be compatible with the limitations concerning the availability of devices and materials present in space missions. Three-dimensional (3D) bioprinting (BP) might become a solution for both demands, as it allows the manufacturing of multicellular, complex and 3D tissue constructs, which can serve as models in basic research as well as transplantable skin grafts. The perspective article provides an overview of the state of the art of skin BP and approach to establish this additive manufacturing technology in space. In addition, the several advantages of BP for utilization in future manned space missions are highlighted.

## Introduction

The human body, in general, has a huge capacity for wound healing; although, it depends on the general health situation of the individual, the extent of damage suffered by tissues, and the capacity of those cells to multiply. Based on the proliferation capacity of the cells, we can find labile (always in renewal, as skin or bone), stable (able to regenerate when damaged, as liver or kidney), and permanent (almost no regeneration, as cardiac and skeletal muscle) tissues (Paul and Sharma, 2021). Skin, which is the outer covering of the body, is a labile tissue, so cells are under constant active division, replacing the damaged or aged layers continuously. Withal, when the injury is too big as in extensive or severe burns [e.g., in case of partial thickness burns >20% of the total body surface area in an adult, or 10% in children and seniors (European Burns Association., 2017)], the remaining cells are not capable of closing the wound. In these cases, it becomes necessary to transplant healthy skin from other areas of the body (autograft) and utilize artificial skin substitutes or even skin from a human donor (allograft). The optimal option is always the use of autologous cells or tissues, as immunological rejection is avoided. However, harvesting skin for autologous transplantation or isolation of cells leads to the formation of additional lesions.

In the last decades, new materials and methodologies have been developed for the fabrication of improved skin substitutes, including the utilization of emerging technologies (Tottoli et al., 2020; Dai et al., 2020; Tavakoli and Klar 2021). In this regard, applicable also for other tissues as bone or cartilage, three-dimensional (3D) bioprinting (BP) allows the production of complex, cellularized constructs that may overcome the limitations present in the classical methods of tissue engineering, where commonly a prefabricated scaffold is seeded with cells. BP is an additive manufacturing technology and can be seen as a specific type of tissue engineering method. In contrast to the conventional approach, in BP, the live cells are printed together with suitable biomaterial(s) so that a tissue-like construct can be fabricated in one process step.

All the above mentioned is effective under Earth conditions. But, when considering a long-term space exploration mission, as travelling to Mars or settlements on the Moon, it becomes necessary to consider pathologic wound healing due to altered gravity and radiation effects (Grimm et al., 2020). This, in addition to the deconditioning caused by such environmental factors over a prolonged period, makes it necessary to find new strategies for wound healing in space, both concerning advanced research models and for medical treatments.

In this article, a perspective from the BP point of view for healing of skin wounds in space is described, along with a brief review of selected studies about BP of the skin. As the international space agencies have started to implement BP devices at the International Space Station (ISS) (Cubo-Mateo et al., 2020), such approaches are no longer science fiction but will become feasible soon.

## Three-dimensional Bioprinting

**FIGURE 1:**
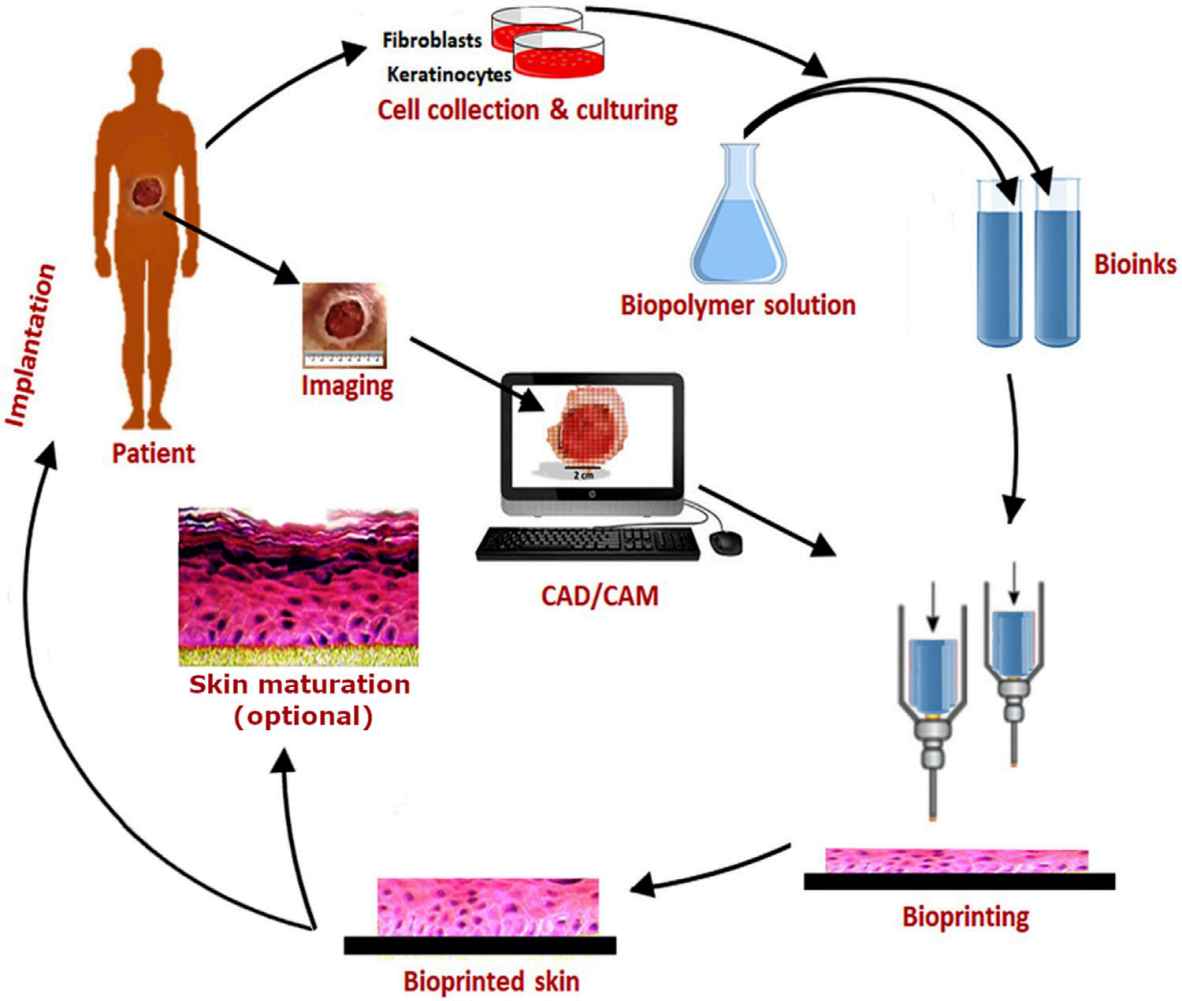
Schematic showing the workflow of BP using the example of extrusion BP of skin utilizing two different cell types (fibroblasts and keratinocytes) and bioinks. Original image taken with permission from Augustine (2018) was adapted.

BP is a type of additive manufacturing in which live cells are included directly in the printing process, mostly in combination with biomaterials. Two main technologies have to be distinguished, extrusion and inkjet (drop-on-demand) BP. In extrusion BP, continuous strands consisting of cells that are suspended in a gellable, viscous liquid called bioink are deposited in a layer-by-layer fashion so that easily 3D constructs can be generated (Askari et al., 2021). In contrast, in inkjet BP, discrete droplets containing single or few cells, small cell aggregates, or even organoids without or with the addition of biomaterial components are deposited (Gudapati et al., 2016). In case of utilization of cell aggregates or spheroids, the term bioassembly is also common for this method (Mironov et al., 2009). The main advantage of BP compared to conventional tissue engineering is the opportunity to deposit different cell types along with specific biomaterials with high spatial resolution. For multilayered tissues like the skin, consisting of different cell types, this facilitates the fabrication process significantly. The whole field of BP has been tremendously developing since a couple of years, and the current state of the art is being described and reviewed continuously (Shapira and Dvir, 2021; Zhang et al., 2021). BP technologies also offer new opportunities for process automation and standardization which is beneficial for translation into clinical applications, as well as for utilization in isolated environments with strictly limited facilities, like in space flight. The principle of BP is illustrated in Figure 1 using the example of extrusion skin BP.

## Current Status of Skin Bioprinting

Human skin is consisting of two main layers, the epidermis as the outermost part and the dermis. While the epidermis contains only one cell type, the keratinocytes, the dermis is dominated by fibroblasts. However, the dermis also contains blood vessels and nerves and several skin appendages like sebaceous and sweat glands – and therefore numerous other cell types. If skin patches are produced for wound healing applications in most cases only fibroblasts and keratinocytes are included to keep the fabrication simple. The skin is one of the human tissues for which replication utilizing BP has been intensively explored, and several studies have been published that describe approaches for the fabrication of artificial skin of variable complexity (Perez-Valle et al., 2020; Tan et al., 2021; Weng et al., 2021). For skin BP, several methods including extrusion and inkjet printing are applied, and also additional BP technologies like laser-induced forward transfer (LIFT) (Koch et al., 2012). Table 1 summarizes selected studies which describe BP of multilayer skin constructs, consisting of (at least) a dermal and epidermal compartment. This list shall provide a concise overview about the variety of approaches for using BP to fabricate skin models. One has to distinguish two fundamentally different approaches in skin BP: one is the fabrication of skin-like grafts in the lab, which commonly are further cultivated *in vitro* before implantation onto a wound, whereas the other describes the direct deposition of cells and materials onto the lesion of the patient (or experimental animal). The latter has been defined as “*in situ*” or “*in vivo* BP” and can be performed with a robotic arm device or just a handheld deposition system (Singh et al., 2020).

**TABLE 1:** Selected skin BP approaches performed under standard gravity conditions. No. 1–5 describe conventional studies (*in vitro* BP), No. 6–8 examples for *in situ* skin BP.

No	References	Dermal bioink	Fibroblasts (FB) density	Epidermal bioink	Keratinocytes density (KC)	Comments
1	Lee et al. (2009)	Rat tail type I collagen, diluted in DPBS	1 × 10^6^ primary hFB/mL of bioink	Collagen type I diluted in DPBS	1 × 10^6^ hKC/mL of hydrogel	Drop-on-demand BP. Separate printing of biomaterial components and suspended cells
2	Koch et al. (2012)	MatriDerm™, rat tail type I collagen	1.5 × 10^6^ NIH-3T3 FB, resuspended in 1 × DMEM/Ham’s F12 medium	Rat tail type I collagen	Same density than for FB. HaCaT KC	LIFT BP. Almost no support material between cells. High accuracy, important for future vascular network forming
3	Cubo et al. (2017)	Human plasma from blood (+tranexamic acid, CaCl_2_, and NaCl 0.9%) with embedded primary hFB	1 × 10^3^ hFBs/cm^2^ (for a 3-mm thick gel: 3 × 10^3^ FB/mL of bioink)	None, human primary KC suspended in KC medium	>1.5 × 10^4^ hKCs/cm^2^	Extrusion BP. Includes *in vivo* study (nude mice). Layers cannot be piled up, too liquid. Quick patient treatment possible: final cell expansion *in situ* inside the plasma hydrogel and *in vivo* maturation. Natural wound healing process
4	Baltazar et al. (2020)	Rat tail type I collagen, FBS and reconstitution buffer (embedded FB, and in some cases, 7 × 10^5^/ml human EC with or without 3.5 × 10^5^/ml human PC)	7.0 × 10^5^ hFB/mL of bioink	KC growth medium and skin differentiation supplemented medium	2 × 10^6^ hKC/mL of media	Extrusion BP. Includes *in vivo* study (nude mice). The PC in the dermal bioink associate with EC-lined vascular structures and appear to improve KC maturation
5	Admane et al. (2019)	5% silk fibroin and 5% gelatin	2 × 10^6^ hFB/mL of bioink	5% silk fibroin and 5% gelatin	5 × 10^6^ hKC/mL of bioink	Extrusion BP. Good reproduction of the dermal–epidermal interface and in-depth gene expression analysis
***In situ* skin BP approaches**						
6	Skardal et al. (2012)	Fibrinogen and type I rat tail collagen	No FB; 1.66 × 10^7^ human AFS and MSC/mL	No epidermal layer	No KC	Drop-on-demand BP, directly onto a wound generated in nude mice. Use of MSC and AFS beneficial to wound healing and immune privileged even though they originated from an allogenic source
7	Hakimi et al. (2018)	Bovine fibrinogen, sodium hyaluronate, type I rat tail collagen dissolved in PBS	0.5 × 10^6^ hFBs/mL	Bovine fibrinogen, sodium hyaluronate	1.25 × 10^6^ hKC/mL of hydrogel	Handheld BP, directly onto wounds generated in nude mice and Yorkshire pigs*.* Quick wound coverage: 0.3–1.6 cm^2^/s. Normal reepithelization and wound contraction
8	Albanna et al. (2019)	25 mg/ml bovine fibrinogen and 1.1 mg/ml rat tail type I collagen	3.75 × 10^7^ FB/mL of hydrogel (allogenic: human, autologous: murine and porcine FB)	25 mg/ml fibrinogen and 1.1 mg/ml collagen type I	7.5 × 10^7^ kC/ml of hydrogel (allogenic: human, autologous: murine and porcine KC)	Ink-jet BP, directly onto wounds generated in nude mice and specific pathogen-free Yorkshire pigs*.* Wounds treated using *in situ* skin BP demonstrated faster wound closure compared to untreated and matrix-treated group

Abbreviations: h = human, FB = fibroblasts, KC = keratinocytes, EC = endothelial cells, PC = placental pericytes, hDPC = human dental pulp cells, AFS = amniotic fluid–derived stem cells, MSC = bone marrow–derived stem cells, LIFT = laser-induced forward transfer, DPBS = Dulbecco’s phosphate-buffered saline.

In the studies included in Table 1, the skin-like constructs that are bioprinted are simplified compared to the native tissue. For printing the dermal layer commonly only fibroblasts are utilized and for the epidermal layer keratinocytes. However, few studies have already described the inclusion of additional cell types like preadipocytes (for providing a hypoderm-like third layer for the treatment of full-thickness skin defects or burns) or endothelial cells (to support fast vascularization of the constructs). Ng and co-workers have demonstrated bioprinting of pigmented skin by inclusion of melanocytes (Ng et al., 2018). Due to the fact that the extracellular matrix (ECM) is the largest component of the dermis, constituting over 70% of this tissue (Widgerow et al., 2016), the fibroblasts are in most cases applied as part of a bioink, consisting of hydrogel-forming (bio)polymer solutions, whereas the keratinocytes are bioprinted both with the matrix components or just as a cell suspension on top of the dermal layer.

A variety of bioinks have been investigated and applied so far for BP of the dermal layer, and most of them are based on single biopolymers or blends (Perez-Valle et al., 2020; Masri and Fauzi, 2021). As native ECM of the dermis mainly consists of collagen type I, this biopolymer is an obvious suitable choice and also many other types of biopolymers, including gelatin, fibrin, chitosan, and alginate have been applied successfully. As the scaffolds in conventional tissue engineering, the bioinks in BP shall provide only temporary support, being replaced by the natural ECM synthesized by the embedded cells (i.e. fibroblasts in case of the dermal layer) or cells invading the implanted graft from the surrounding tissue over time.

## Wound and SKIN Healing in Space

The skin provides the outer covering of the body and therefore has a protective function: it avoids excessive water loss and prevents pathogens from entering the organism. It also regulates body temperature and contains several types of glands and sensors (nerve endings) that allow us to feel objects and to secret metabolites. With increasing age, the human skin becomes more fragile and thin and requires longer periods to heal from injuries (Dyer and Miller, 2018). In space, environmental factors such as microgravity and radiation can have a severe impact on different tissues, and the effect can be quickly seen on the skin, bones and cartilages, muscles, and some internal organs like the heart (Afshinnekoo et al., 2020).

Astronauts lose more skin cells (keratinocytes) in space than on the Earth, and their skin ages faster during space flight; a common complaint of astronauts is cracking skin and rashes or itchiness. Apart from that, a thinning of the skin and increased sensitivity combined with delayed healing of wounds and an increased tendency to skin infections have been reported during and after long-term stays in space. These data have been obtained from three experiments carried out at the ISS regarding tissue development of humans and mice in space (Lademann and Fluhr, 2008; Tronnier et al., 2008; König et al., 2015; Neutelings et al., 2015; Braun et al., 2019). More details and the newest findings regarding wound and skin healing in space are described elsewhere in the present special issue of which this article is part of.

### Bioprinting of Skin for Wound Healing in Space

While it is already widely accepted that additive manufacturing using nonbiological materials will play a crucial role in the further development of space flight (Ghidini 2018), the international space agencies have started to become interested in BP too. The authors recently have described the relevance of BP for future long-term and far-distant manned space missions, and the current status of the establishment of bioprinters at the ISS in a separate study (Cubo-Mateo et al., 2020). To summarize, the two main objectives for using BP in space are: on the one hand, the opportunity to fabricate complex, multicellular, and 3D tissue models to investigate the effects of space conditions on cells and tissues on-site; on the other hand, there is the hope that BP once could provide tissue constructs for the medical treatment of injured or diseased astronauts. As skin is the tissue being mostly exposed to the environment, it also has the highest risk of being injured. In a study about traumatic injuries during long-duration spaceflight, those of the skin were reported to be among the most frequent and likely ones (Kirkpatrick et al., 2009). Together with the fact of impaired wound healing in space, the ability to be able to bioprint skin tissue might be of crucial importance for future manned space missions like lunar settlements or Mars exploration. BP therefore can help to increase the autonomy of the crew on long-term missions, and as the bioprinted constructs are already cellularized and ready-to-use, the wound healing (even when impaired because of the environmental factors) can be improved and accelerated.

It has been already demonstrated that human cells can be grown under altered gravity conditions thanks to rotary devices that allow the generation of 1G conditions in space (as rotatory vessels or centrifuge chambers) (Grimm et al., 2020). Therefore, if the BP technology can be adapted to work under space conditions (which is quite likely), the previous cell expansion and the posterior maturation of the printed tissues could be managed inside such devices. As maturation of the epidermal layer of bioprinted skin requires contact to air, cultivation under simulated gravity would help in providing the necessary air–liquid interface.

For applications in space flight, accessibility of all components is of utmost importance because of the very limited payload capacities. Therefore, it would be obvious to utilize autologous cells, isolated from an injured astronaut in case of the need for BP of a tissue for medical application. In addition, this would lead to an autologous skin graft, which is being preferred because of immunological compatibility. An interesting possibility would be to create cell banks for (and from) the crew with healthy cells of the most interesting tissues, including mesenchymal stem cells, taken even before launch from the Earth. Also, 3D models of their organs and bone structures could be archived from CT/MRI (computed tomography/magnetic resonance imaging) data sets. Regarding materials, also the other components for bioink preparation could be provided on-site: this can be biopolymers like alginate, isolated from algae which could be cultivated in space—or constituents of human blood like whole plasma or fibrinogen which again could be derived from the astronauts themselves. It already could be demonstrated that human plasma and fibrinogen are suitable bioink components for skin BP (Cubo et al., 2017; Albanna et al., 2019).

Also the *in situ* BP approach, briefly explained above, is of interest for applications in space flight as it would provide a fast and easy opportunity to support wound healing without the necessity to further cultivate and mature the bioprinted constructs prior to deposition. Currently, the space company OHB is developing for the German Space Agency at DLR a handheld skin BP device, based on the extrusion printing principle, which shall be sent to the ISS at the end of the year 2021 for evaluation (DLR, 2020).

## Discussion and Conclusions

BP is a tremendously developing field of research. Several BP technologies and a huge variety of suitable biomaterials for the printing of live cells have been developed and are available (Chimene et al., 2016; Gungor-Ozkerim et al., 2018; Li et al., 2020). Skin BP is one of the applications being already investigated in depth, and numerous studies have proven the applicability in principle, utilizing various BP technologies, cell types, and biomaterials. Within the available technologies of the additive manufacturing family, the most developed methods for BP are based on extrusion or drop-on-demand printing. Between these two, probably extrusion BP is more mature and easier to translate first into space because it presents a more basic mechanism and requires a smaller number of cells, as it works with cell-material suspensions. Therefore, also less time is required for cell expansion prior to the BP process when the cells are “diluted” in the respective bioinks. In addition, in extrusion BP more viscous bioinks can be utilized compared to inkjet BP that would present higher stability while printing in microgravity and for the post-printing process steps. Finally, such pasty cell-laden bioinks can be designed to be quite sticky which would facilitate the layer-by-layer assembly process under microgravity conditions and also allows *in situ* BP applications, i.e., direct deposition of such bioinks onto skin wounds (Cubo-Mateo et al., 2020).

The main advantages of utilizing BP technologies for fabrication of transplantable skin grafts can be summarized as follows:

• Compared to conventional tissue engineering approaches, BP can be done in a semi-automated manner which is beneficial when applied during space flight,
• Again compared to TE, the fabrication process in BP is faster and simpler as biomaterials and cells are deposited together whereas in TE first a scaffold has to be made which then is seeded with the different cell types, one after the other,
• BP provides better control concerning the dimensions and internal structure (e.g., thickness of dermal and epidermal layers) of the skin grafts,
• For every cell type included in the BP process, optimal biomaterials can be selected, e.g., mimicking the respective local ECM composition,
• In principal, BP allows manufacturing of complex tissue equivalents by integration of hair follicles, melanocytes, eccrine sweat and sebaceous glands, etc., or cell types supporting fast vascularization.

Initially, BP will be applied in space for the fabrication of more complex, multicellular, and 3D tissue models for research purposes, which mimic the native human tissues better than conventional cell cultures used so far. For this purpose, the international space agencies already have started to install such devices at the ISS. However, there is hope that the technologies will develop further so that once BP might be able to support medical treatments and help astronauts on far-distant space missions or in extraterrestrial human settlements, e.g., on the Moon, to survive. As the skin is the tissue mostly at risk to become injured and wound healing is known to be impaired in space, further developments of BP of the skin is believed to be an important topic.

## Data Availability

The original contributions presented in the study are included in the article/Supplementary Material; further inquiries can be directed to the corresponding authors.
